# Selective phosphorylation of threonine residues defines GPR84–arrestin interactions of biased ligands

**DOI:** 10.1016/j.jbc.2022.101932

**Published:** 2022-04-12

**Authors:** Sara Marsango, Richard J. Ward, Laura Jenkins, Adrian J. Butcher, Zobaer Al Mahmud, Louis Dwomoh, Falko Nagel, Stefan Schulz, Irina G. Tikhonova, Andrew B. Tobin, Graeme Milligan

**Affiliations:** 1 The Centre for Translational Pharmacology, Institute of Molecular, Cellular and Systems Biology, College of Medical, Veterinary and Life Sciences, University of Glasgow, Glasgow, United Kingdom; 2 Department of Clinical Neurosciences, University of Cambridge, Cambridge, United Kingdom; 3 7TM Antibodies GmbH, Jena, Germany; 4 Institute of Pharmacology and Toxicology, University Hospital Jena, Jena, Germany; 5 School of Pharmacy, Medical Biology Centre, Queen's University Belfast, Belfast, United Kingdom

**Keywords:** GPR84, G protein–coupled receptor phosphorylation, GRK2/3, arrestin recruitment, phospho-site specific antisera, 2-HTP, 2-(hexylthio)pyrimidine-4,6-diol, 6-OAU, 6-n-octylaminouracil, λ-PPase, Lambda protein phosphatase, BRET, bioluminescence resonance energy transfer, BSA, bovine serum albumin, CHO, Chinese hamster ovary, cmp101, compound 101, compound 837, 3-((5,6-Diphenyl-1,2,4-triazin-3-yl)methyl)-1H-indole, Ct, C-terminal tail, DL-175, (3-(2-((4-chloronaphthalen-1-yl)oxy)ethyl)pyridine 1-oxide), Dox, doxycycline, EL2, second extracellular loop, eYFP, enhanced Yellow Fluorescent Protein, GLPG1205, 9-cyclopropylethynyl-2-((S)-1-[1,4]dioxan-2-ylmethoxy)-6,7-dihydropyrimido[6,1-a]isoquinolin-4-one, GPCR, G protein–coupled receptor, GRK, G protein–coupled receptor kinase, GTPγS, guanosine 5′-O-[gamma-thio]triphosphate, HBSS, Hank’s balanced salt solution, HEK, human embryonic kidney, IL3, third intracellular loop, MCFAs, medium-chain fatty acids, pm, phosphomutant, PSB-16671, di(5,7-difluoro-1H-indole-3-yl)methane

## Abstract

GPR84 is an immune cell–expressed, proinflammatory receptor currently being assessed as a therapeutic target in conditions including fibrosis and inflammatory bowel disease. Although it was previously shown that the orthosteric GPR84 activators 2-HTP and 6-OAU promoted its interactions with arrestin-3, a G protein–biased agonist DL-175 did not. Here, we show that replacement of all 21 serine and threonine residues within i-loop 3 of GPR84, but not the two serines in the C-terminal tail, eliminated the incorporation of [^32^P] and greatly reduced receptor–arrestin-3 interactions promoted by 2-HTP. GPR84 was phosphorylated constitutively on residues Ser^221^ and Ser^224^, while various other amino acids are phosphorylated in response to 2-HTP. Consistent with this, an antiserum able to identify pSer^221^/pSer^224^ recognized GPR84 from cells treated with and without activators, whereas an antiserum able to identify pThr^263^/pThr^264^ only recognized GPR84 after exposure to 2-HTP and not DL-175. Two distinct GPR84 antagonists as well as inhibition of G protein–coupled receptor kinase 2/3 prevented phosphorylation of pThr^263^/pThr^264^, but neither strategy affected constitutive phosphorylation of Ser^221^/Ser^224^. Furthermore, mutation of residues Thr^263^ and Thr^264^ to alanine generated a variant of GPR84 also limited in 2-HTP–induced interactions with arrestin-2 and -3. By contrast, this mutant was unaffected in its capacity to reduce cAMP levels. Taken together, these results define a key pair of threonine residues, regulated only by subsets of GPR84 small molecule activators and by GRK2/3 that define effective interactions with arrestins and provide novel tools to monitor the phosphorylation and functional status of GPR84.

GPR84 is a nominally orphan G protein–coupled receptor (GPCR) that can be activated by both medium-chain fatty acids (MCFAs) and a range of synthetic ligands that act as either orthosteric or allosteric activators (1, 2, 3). The pattern of expression across a variety of both peripheral and central immune cells (4, 5, 6, 7), and that mRNA and protein corresponding to the receptor are strongly upregulated in response to proinflammatory challenges (6, 7), has promoted interest in GPR84 as a therapeutic target in a range of areas (1, 2). These include both ulcerative colitis and idiopathic pulmonary fibrosis (1, 8). Although focus for both these indications has been on the development and assessment of antagonist ligands (1, 3, 8), it has been suggested that agonists of GPR84 might also find use in, for example, the treatment of atherosclerosis (9). No matter the disease area, understanding of the extent of upregulation of the receptor in disease settings and the degree of activation of the receptor in such situations is vital. Progress in the production and characterization of a [^3^H]radiolabeled agonist (10) and [^3^H]radiolabeled antagonists (7, 8, 11) can potentially provide insights into the former of these questions, but other reagents and biosensors will be needed to probe the degree of receptor activation.

A broadly applicable feature of GPCRs following agonist occupancy is that phosphorylation of various hydroxy-amino acids within intracellular sequences of the receptor is stimulated and this promotes the effectiveness of interactions of the receptor with arrestin isoforms (12, 13). This can limit the ability of the receptor to interact with G proteins and hence restrict signaling. It can also potentially promote other distinct signaling cascades and generally promotes movement of the receptor away from the cell surface and into intracellular locations. The patterns and locations of the amino acids that become phosphorylated in an agonist-dependent manner have been extensively mapped for a range of GPCRs (14, 15, 16, 17, 18), and both mass spectrometry and mutagenesis have contributed to this effort. Information taken from such studies can then be used to generate antisera able to identify specifically individual phosphorylated amino acids or groups of such amino acids (14, 15, 16, 19, 20). These can then potentially be used as surrogate reagents to detect and demonstrate the extent of receptor activation *in situ*.

Among ligands that activate GPR84, the recently described molecule 3-(2-((4-chloronaphthalen-1-yl)oxy)ethyl)pyridine 1-oxide (DL-175) is reported to be functionally “biased,” in that, although a potent regulator of G protein–mediated signaling, it is not effective in promoting arrestin interactions (21) and has distinct characteristics in signaling compared with the agonist 6-n-octylaminouracil (6-OAU) (21). Because we anticipated that DL-175 and 6-OAU might well produce a different pattern or extent of phosphorylation of GPR84, to obtain a fuller understanding of such potential variation caused by GPR84 activators, herein in addition we have assessed both 2-(hexylthio)pyrimidine-4,6 diol (2-HTP) as a potent orthosteric activator (7) and the allosteric activator PSB-16771 (22). This is a more potent analogue of the originally defined allosteric activator of GPR84, 3,3′-diindolylmethane (23).

We show that, although both 2-HTP and 6-OAU produce effective phosphorylation of human GPR84 at residues within the third intracellular loop, neither DL-175 nor PSB-16771 does so. Moreover, although each of 2-HTP, 6-OAU, and DL-175 acts as orthosteric agonists of GPR84, we use homology modeling and mutagenesis to provide a molecular basis for their differences in function and bias. In addition, based on mass spectrometry, we identify residues in the third intracellular loop of GPR84 that are phosphorylated constitutively. Production of antisera that identify specific sites of both constitutive and agonist-regulated phosphorylation allowed us to demonstrate that phosphorylation at amino acids Thr^263^ and/or Thr^264^ is required to generate highly effective interactions with arrestin-2 and arrestin-3 and that these amino acids become phosphorylated in a GRK2/3-dependent manner. By contrast, neither Ser^221^ nor Ser^224^, which are constitutively phosphorylated and not affected by inhibition of GRK2/3, plays a major role in arrestin-2 or arrestin-3-interactions. However, the phosphorylation status of neither of these sets of amino acids impacts on the ability of the receptor to regulate cellular levels of cAMP. Of importance, as GPR84 is markedly upregulated in a wide range of immune cells and in a variety of proinflammatory settings (4, 5, 6) the activation status detection of GPR84 by the agonist-regulated antiserum may provide an important biosensor in model systems of GPR84 regulation and function.

## Results

**Figure 1:**
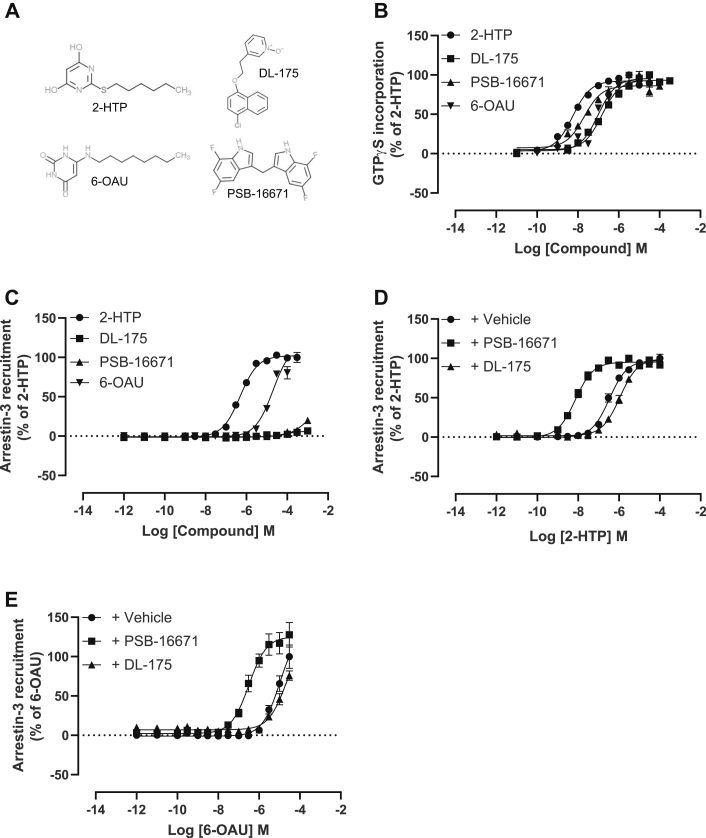
**Both orthosteric and allosteric agonists promote G protein activation by human GPR84, but only a subset promote interactions with arrestin-3.** The ability of varying concentrations of each of 2-HTP, 6-OAU, PSB-16671, and DL-175 (structures shown in *A*) to promote G protein activation was measured *via* binding of [^35^S]GTPγS in membranes generated from Flp-In TREx 293 cells induced to express hGPR84-eYFP (*B*). The same four ligands were used to measure interactions between hGPR84-eYFP and arrestin-3 (*C*). In such arrestin-3 interaction studies PSB-16671 (1.3 × 10^−5^ M) increased the observed potency of 2-HTP and 6-OAU, but DL-175 (9.8 × 10^−5^ M) did not (*D* and *E*). One-way ANOVA followed by Dunnett's multiple comparisons test. Data are means ± SEM. n ≥3.

To study mechanisms of signal transduction induced by activation of the proinflammatory GPCR GPR84 we selected a pair of orthosteric agonists, 2-HTP (7) (also designated “compound 1” (24), or ZQ-16 (25)) and 6-OAU (5, 26), as well as the allosteric activator PSB-16671 (7, 22) (Fig. 1*A*). In addition, we selected DL-175, a compound optimized following a virtual screen and reported to display marked G protein signaling compared with arrestin-interaction bias at GPR84 (21) (Fig. 1*A*). To initiate studies, we expressed stably in Flp-In TREx 293 cells human GPR84 with enhanced Yellow Fluorescent Protein linked in-frame to the intracellular C-terminal tail of the receptor (hGPR84-eYFP) (24). This cell system allows doxycycline-induced expression of constructs located at the Flp-In TREx locus (27). GPR84 is known to be able to interact with pertussis toxin–sensitive members of the G_i_-G protein family (1, 4). In membranes prepared from such doxycycline-induced cells the ability of each ligand to cause activation of G_i_-G proteins was measured *via* [^35^S]GTPγS binding assays. Each compound promoted an increase in binding of [^35^S]GTPγS in a concentration-dependent fashion with rank order 2-HTP > PSB-16671 > 6-OAU = DL-175 (Fig. 1*B*), and each ligand displayed similar efficacy at maximally effective concentrations (Fig. 1*B*). Activation of GPR84 with certain, but not all, ligands is reported to promote interaction with arrestin isoforms (21, 28). When hGPR84-eYFP was transiently coexpressed in human embryonic kidney (HEK) 293T cells with arrestin-3 fused to nanoluciferase, 2-HTP effectively promoted proximity and potential interactions between the receptor and this arrestin construct (Fig. 1*C*). 6-OAU was also able to promote interactions between hGPR84-eYFP and arrestin-3-nanoluciferase. Once more 2-HTP was substantially more potent than 6-OAU, although it was not possible to add 6-OAU at sufficiently high concentrations to allow full concentration–response curves to be defined (Fig. 1*C*) because both compounds displayed substantially lower potency than in the [^35^S]GTPγS binding studies (compare Fig. 1, *B* and *C*). In contrast, DL-175 did not promote measurable interactions between hGPR84-eYFP and arrestin-3-nanoluciferase at any concentration that was practical to assess (Fig. 1*C*) and neither did PSB-16671 (Fig. 1*C*). However, as anticipated for a positive allosteric modulator at GPR84 (7, 22) PSB-16671 substantially increased (*p* < 0.0001) the potency of 2-HTP in such arrestin-3 interaction studies (Fig. 1*D*), and this was also the case for 6-OAU (*p* < 0.01) (Fig. 1*E*). By contrast, coaddition of DL-175 did not increase the observed potency of 2-HTP (Fig. 1*D*) or 6-OAU (Fig. 1*E*).

**Figure 2:**
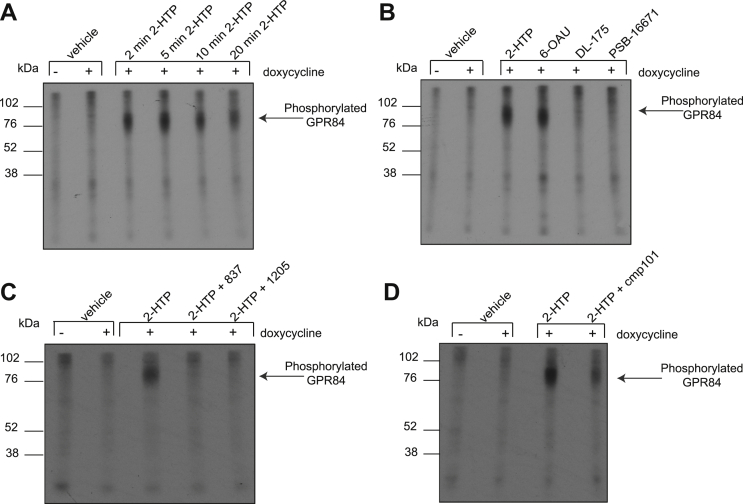
**A subset of GPR84-activating ligands promote phosphorylation of the receptor.** Flp-In TREx 293 cells induced to express hGPR84-eYFP were incubated with [^32^P] orthophosphate and subsequently with various ligands. Lysates from these cells were immunoprecipitated with an anti-GFP antiserum, resolved by SDS-PAGE, and exposed to X-ray film. *A*, cells were treated with vehicle or with 2-HTP (3.7 × 10^−6^ M) for the times indicated. Cells were treated with 2-HTP (3.7 × 10^−6^ M), 6-OAU (6.4 × 10^−5^M), DL-175 (9.8 × 10^−5^M), or PSB-16671 (1.3 × 10^−5^ M) for 5 min (*B*). Cells were treated with 2-HTP and the GPR84 antagonists GLPG1205 (1205) or compound 837 (each at 1 × 10^−5^M) (*C*). Cells were treated with 2-HTP following pretreatment with the GRK2/3 inhibitor compound 101 (cmp101) (1 × 10^−5^ M) (*D*). Representative experiments are displayed.

Interaction of a GPCR with an arrestin is often dependent upon phosphorylation of the receptor that occurs subsequent to agonist occupancy of the receptor (12). To examine this directly we labeled Flp-In TREx 293 cells induced to express hGPR84-eYFP with [^32^P] orthophosphate and then treated the cells with vehicle or a concentration of 2-HTP (3.7 × 10^-6^ M) determined to produce an EC_90_ effect in the arrestin-3 interaction BRET assay. Lysates of these cells were immunoprecipitated with an anti-Green Fluorescent Protein (GFP) antiserum (that also identifies eYFP) and then resolved by SDS-PAGE. After drying, such gels were subsequently exposed to X-ray film. No substantial incorporation of [^32^P] was detected in vehicle-treated samples. However, clear incorporation of [^32^P] into polypeptide(s) with molecular mass corresponding to some 80 kDa was observed following treatment with 2-HTP, with close to maximal incorporation of [^32^P] achieved within 5 min of agonist exposure (Fig. 2*A*). 6-OAU (6.4 × 10^−5^ M) also effectively promoted incorporation of [^32^P] into hGPR84-eYFP (Fig. 2*B*). Because neither DL-175 nor PSB-16671 produced effective interactions of hGPR84-eYFP with arrestin-3, and therefore no EC_90_ concentration could be defined, for [^32^P]-phosphorylation studies we employed concentrations of these ligands that were 100 times greater than their EC_90_ concentrations in [^35^S]GTPγS binding studies on the basis that, as shown earlier, 2-HTP and 6-OAU were to this extent less potent in the arrestin-3 interaction studies than in [^35^S]GTPγS binding studies. However, even at these concentrations, no significant incorporation of [^32^P] into hGPR84-eYFP was observed in samples treated with either DL-175 (9.8 × 10^−5^ M) or PSB-16671 (1.3 × 10^−5^ M) (Fig. 2*B*). As expected, the ability of 2-HTP to promote phosphorylation of hGPR84-eYFP was prevented by coincubation with either of two chemically distinct GPR84 antagonists, GLPG1205 (8) and compound 837 (11) (each at 1 × 10^−5^ M) (Fig. 2*C*). Because agonist-mediated phosphorylation of many GPCRs reflects interactions with members of the GRK family we assessed the effect of the GRK2/3 inhibitor compound 101 (1 × 10^−5^ M) (29, 30). [^32^P] phosphorylation of hGPR84-eYFP in response to 2-HTP was greatly reduced by pretreatment of the cells with compound 101 (Fig. 2*D*) consistent with a major role for GRK2 and/or GRK3.

**Figure 3:**
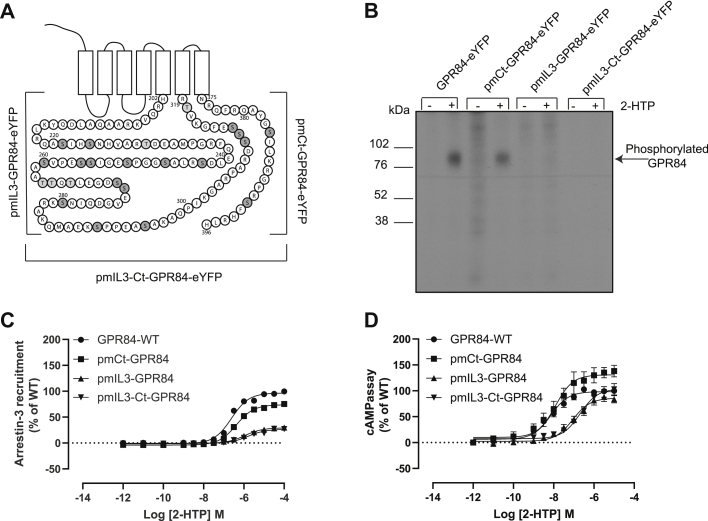
**Removal of potential sites of phosphorylation in the third intracellular loop but not the C-terminal tail prevents agonist-induced phosphorylation of human GPR84 and limits interactions with arrestin-3.** All serine and threonine residues in the third intracellular loop (pmIL3), the C-terminal tail (pmCt), or both the third intracellular loop and the C-terminal tail (pmIL3-Ct) of human GPR84-eYFP were converted to alanine (*A*). Following stable expression of each of these in Flp-In TREx 293 cells and induction of expression by exposure to doxycycline, cells were labeled with [^32^P] orthophosphate and then treated with 2-HTP (3.7 × 10^−6^ M). Subsequent steps were as in Figure 2 (*B*). Wildtype hGPR84-eYFP and the mutants described in **a** were employed in arrestin-3 interaction studies using varying concentrations of 2-HTP (*C*). 2-HTP-mediated regulation of cAMP levels of wildtype, pmCt, pmIL3, and pmIL3-Ct GPR84 are displayed (*D*). pmIL3- and pmIL3-Ct GPR84 displayed reduced potency for 2-HTP (*p* < 0.001). One-way ANOVA followed by Dunnett's multiple comparisons test. Data are means ± SEM. n ≥ 3.

Agonist occupancy frequently results in phosphorylation of key serine and/or threonine residues in the third intracellular loop (IL3) and/or C-terminal tail (Ct) of GPCRs. Human GPR84 has an extensive IL3 containing 21 serine and threonine residues (Fig. 3*A*). We therefore generated a potentially phosphomutant (pm) form of hGPR84-eYFP in which each of these serine/threonine residues was converted to alanine (pmIL3-hGPR84-eYFP). The C-terminal tail is less complex in this regard and contains only two serine residues (Fig. 3*A*). We also, however, generated a variant of hGPR84-eYFP in which these two residues were converted to alanines (pmCt-hGPR84-eYFP). As a combination we further generated an additional construct in which all 21 serines/threonines in IL3 and the two serines in the Ct were altered to alanine (pmIL3-Ct-hGPR84-eYFP) (Fig. 3*A*). Following stable expression of each of these in Flp-In TREx 293 cells and induction of expression by exposure to doxycycline, further [^32^P] phosphorylation studies were performed following addition of 2-HTP. Alteration of the pair of serines in the Ct had no clear effect on the level of incorporation of [^32^P] (Fig. 3*B*). By contrast, removal of all the potential sites of phosphorylation in IL3 ablated incorporation of [^32^P] in response to 2-HTP (Fig. 3*B*), and this was also the case for the variant in which all potential phosphorylation sites from the Ct and IL3 were converted to alanines (Fig. 3*B*). Although the variant lacking the Ct serine residues showed only a small reduction in maximal effect (*p* < 0.05) and no significant alteration in potency in response to 2-HTP in the arrestin-3-interaction assay compared with wildtype hGPR84-eYFP (Fig. 3*C*), the ability of both pmIL3-hGPR84-eYFP and pmIL3-Ct-hGPR84-eYFP to recruit arrestin-3 in response to 2-HTP was severely (*p* < 0.0001) compromised in both maximal effect and agonist potency (Fig. 3*C*). This suggested that Ser-Thr residues within IL3 control arrestin-3 interactions *via* agonist-induced phosphorylation. By contrast, pmIL3-hGPR84-eYFP and pmIL3-Ct-hGPR84-eYFP both showed similar maximal regulation of forskolin-stimulated cAMP levels in response to 2-HTP as wildtype hGPR84-eYFP, although with somewhat reduced potency (*p* < 0.001) (Fig. 3*D*), and maximally effective concentrations of 2-HTP promoted a somewhat greater reduction of cAMP levels *via* pmCt-hGPR84-eYFP than wildtype hGPR84-eYFP (*p* < 0.05) (Fig. 3*D*).

**Figure 4:**
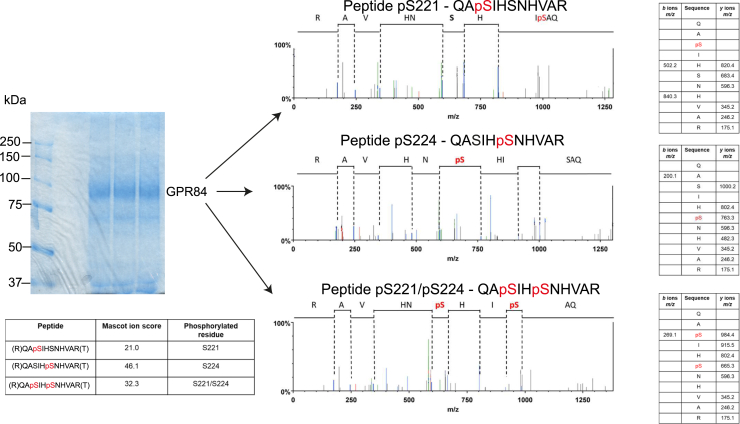
**Mass spectrometry defines sites of constitutive phosphorylation of human GPR84.** GPR84 immunoprecipitated from CHO-K1 cells was subjected to mass spectrometry after tryptic digestion as detailed in Experimental procedures. LC-MS/MS identified Ser^221^ and Ser^224^ as being phosphorylated constitutively. Composite outcomes of a series of independent experiments are combined. Fragmentation tables associated with phosphorylated peptides. Phosphorylated residues are highlighted in *red*. Peptide prob. indicates percentage probability of a correct peptide based on the discriminant score; both are generated by Scaffold software.

**Figure 5:**
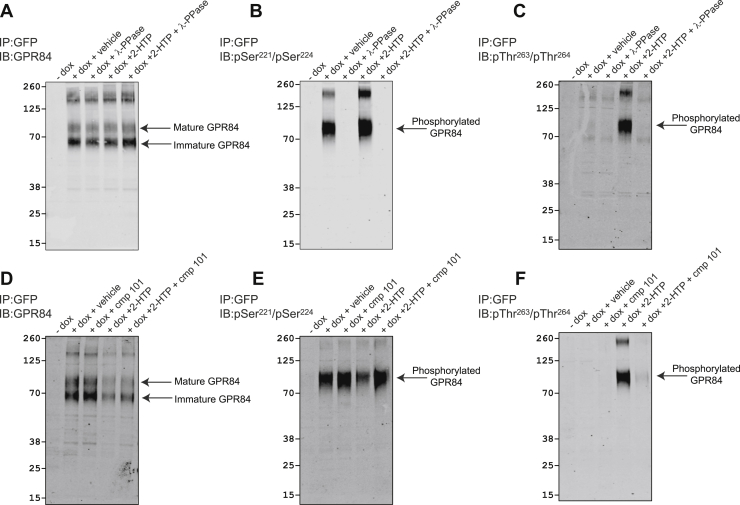
**Characterization of GPR84 phospho-site-specific antisera.** Flp-In TREx 293 cells harboring human GPR84-eYFP (-dox) or induced to express the receptor construct (+dox) were treated with Lambda protein phosphatase (λ-PPase), 2-HTP, and/or compound 101 (cmp101) as indicated. Following enrichment of the receptor construct *via* GFP-trapping and SDS-PAGE immunoblots were performed with the C-terminal GPR84 structural antiserum (*A* and *D*) or the phospho-site-targeted antisera pSer^221^/pSer^224^ (*B* and *E*), and pThr^263^/pThr^264^ (*C* and *F*). Apparent molecular mass markers are shown, and mature and immature forms of GPR84 are highlighted. Illustrative outcomes are displayed. dox, doxycycline.

To gain further and more direct insight into specific residues in the IL3 of hGPR84-eYFP that either become phosphorylated in response to 2-HTP or might be constitutively phosphorylated in the absence of agonist activation we performed a series of mass spectrometry studies on proteins and peptides isolated from both Chinese hamster ovary (CHO)-K1 cells and Flp-In TREx 293 cells stably expressing hGPR84-eYFP (Fig. 4). In both the presence or absence of agonist, both Ser^221^ and Ser^224^, located in IL3, were consistently observed to be phosphorylated (Fig. 4). We also observed various other sites that were modified only in response to 2-HTP. Based on such observations we synthesized peptides encompassing such regions and in which phosphoserine and phosphothreonine replaced the nonmodified hydroxy-amino acids (Table 1). These were used as antigens to generate immune responses in rabbits. Following affinity purification such antisera were used to probe for potential basal and 2-HTP-mediated phosphorylation of these residues in extracts of lysates produced from Flp-In TREx 293 cells induced to express hGPR84-eYFP. Herein we focus on two of these antisera. Samples were enriched *via* a GFP-trap, and eluted material was subjected to SDS-PAGE and immunoblotting. As a control, samples were immunoblotted with an anti-GPR84 structural antiserum raised against the sequence corresponding to amino acids 377 to 396 (Gln-Phe-Arg-Gln-Ala-Tyr-Gly-Ser-Ile-Leu-Lys-Arg-Gly-Pro-Arg-Ser-Phe-His-Arg-Leu-His-COOH) within the intracellular Ct of the human receptor. Such studies with this structural antiserum confirmed turn-on of expression of hGPR84-eYFP by doxycycline treatment, that short-term treatment with 2-HTP did not affect levels of the receptor construct, and that treatment of samples with Lambda protein phosphatase (λ-PPase) to remove all phosphates from the GPR84 receptor did not affect recognition of hGPR84-eYFP by this antiserum (Fig. 5*A*). Of interest, such immunoblots identified each of a dominant 67-kDa polypeptide, a more diffuse set of polypeptides with mobility centered at some 75 kDa, and a set of bands close to twice this apparent molecular mass (Fig. 5*A*). Immunoblotting of equivalent samples with an antiserum anticipated to identify pSer^221^/pSer^224^ showed that hGPR84-eYFP was also identified by this antiserum both with and without pretreatment of cells with 2-HTP (Fig. 5*B*). This is consistent with the mass spectrometry data (Fig. 4) that indicated the receptor to be phosphorylated constitutively at these positions. A key conclusion of these studies was that recognition of hGPR84-eYFP by this antiserum did indeed reflect phosphorylation of one or other, or both, of Ser^221^ and Ser^224^ both with and without pretreatment with 2-HTP, because recognition was lacking in samples treated with λ-PPase to remove phosphate from the protein (Fig. 5*B*). A further key observation was that the form of hGPR84-eYFP that migrated at 67 kDa was not identified by the anti-pSer^221^/pSer^224^ antiserum (Fig. 5*B*). This suggests that this form of the receptor is potentially immature, whereas the diffuse group of polypeptides with mobility of some 75 kDa reflect the mature and likely variably N-glycosylated forms. In addition, the more dominant of the low mobility set of polypeptides was also not identified by the anti-pSer^221^/pSer^224^ antiserum while the more diffuse forms were (Fig. 5*B*). These lower-mobility forms may represent either aggregated mature receptor or, more speculatively, a dimeric form (Fig. 5*B*).

**Table 1:** Peptides used to generate anti-GPR84 antisera

Antiserum name	Peptide
Anti-pSer^221^/pSer^224^ antiserum	LRQA**pS**IH**pS**NHVAR
Anti-pThr^263^/pThr^264^ antiserum	VSAA**pTpT**QTLEG
Anti-GPR84 antiserum (structural antiserum)	QFRQAYGSILKRGPRSFHRLH

By contrast, immunoblotting with an anti-pThr^263^/pThr^264^ antiserum (Fig. 5*C*) only identified hGPR84-eYFP after cell treatment with the agonist 2-HTP. Once more recognition by this antiserum was dependent upon phosphorylation because this was eliminated by sample pre-exposure to λ-PPase (Fig. 5*C*). As for the anti-pSer^221^/pSer^224^ antiserum, the anti-pThr^263^/pThr^264^ antiserum (Fig. 5*C*) also failed to recognize the, potentially immature, 67 kDa form of hGPR84-eYFP. As highlighted earlier the GRK2/3 inhibitor compound 101 was able to substantially block 2-THP-mediated phosphorylation of hGPR84-eYFP. In immunoblot studies, although pretreatment with compound 101 had no effect on recognition of the receptor by either the structural GPR84 antiserum (Fig. 5*D*) or the anti-pSer^221^/pSer^224^ antiserum (Fig. 5*E*), such pretreatment entirely prevented recognition of 2-HTP-activated hGPR84-eYFP by the anti-pThr^263^/pThr^264^ antiserum (Fig. 5*F*).

**Figure 6:**
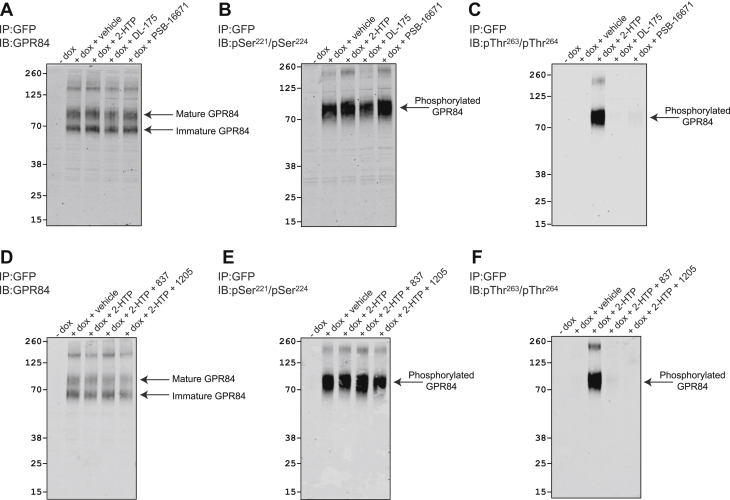
**DL-175 and PSB-16671 do not regulate phosphorylation of specific residues, whereas GPR84 antagonists block 2-HTP-stimulated but not constitutive phosphorylation of GPR84.** + Dox samples as in Figure 5 were treated with 2-HTP, DL-175, or PSB-16671 (*A–C*) or with 2-HTP in the absence or presence of antagonist compounds GLPG1205 (1205) or 837 (*D*–*F*). After enrichment of the receptor construct *via* GFP-trapping and SDS-PAGE immunoblots were performed with the C-terminal GPR84 structural antiserum (*A* and *D*) or the phospho-site-targeted antisera pSer^221^/pSer^224^ (*B* and *E*), and pThr^263^/pThr^264^ (*C* and *F*). Apparent molecular mass markers are shown, and mature and immature forms of GPR84 are highlighted. Representative experiments are displayed. dox, doxycycline.

Treatment with either DL-175 or PSB-16671 did not alter recognition of hGPR84-eYFP by the GPR84 Ct-structural antiserum (Fig. 6*A*). Moreover, treatment with either DL-175 or PSB-16671 did not alter receptor recognition by the anti-pSer^221^/pSer^224^ antiserum, indicating that these ligands do not promote dephosphorylation of these residues (Fig. 6*B*). In agreement with the lack of incorporation of [^32^P] into hGPR84-eYFP in response to either DL-175 or PSB-16671, treatment with these ligands was unable to promote recognition of the receptor by the anti-pThr^263^/pThr^264^ antiserum (Fig. 6*C*). Although treatment with either of the chemically distinct GPR84 antagonists GLPG1205 (8) and compound 837 (11) did not affect recognition of hGPR84-eYFP by the Ct structural GPR84 antiserum (Fig. 6*D*), or recognition by the pSer^221^/pSer^224^ antiserum (Fig. 6*E*), they both fully blocked recognition by the pThr^263^/pThr^264^ antiserum induced by 2-HTP (Fig. 6*F*).

**Figure 7:**
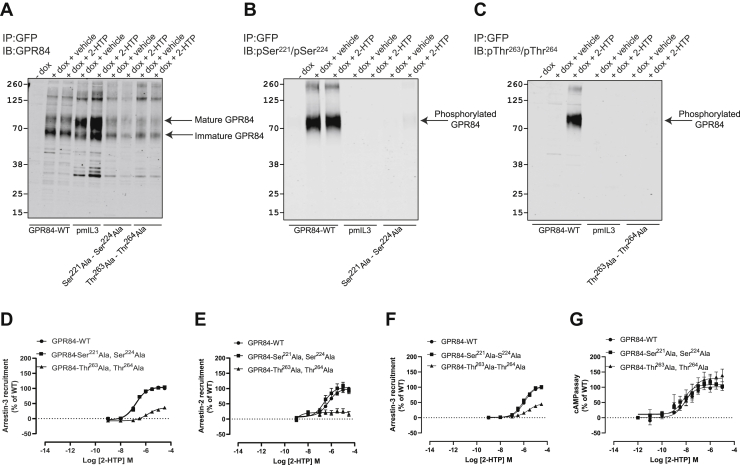
**Phosphorylation of GPR84 Thr**^**263**^**and Thr**^**264**^**is largely responsible for 2-HTP-induced interactions with arrestin-2 and arrestin-3.** Flp-In TREx 293 cells were induced to express wildtype GPR84, pmIL3-GPR84, Ser^221^Ala, Ser^224^Ala-GPR84, or Thr^263^Ala, The^264^Ala-GPR84, each C-terminally tagged with eYFP, as noted. Following treatment of cells with or without 2-HTP, immunocapture, and SDS-PAGE as in Figures 5 and 6 samples were immunoblotted with the C-terminal tail structural anti-GPR84 antiserum (*A*), pSer^221^/pSer^224^ (*B*), or pThr^263^/pThr^264^ (*C*). Wildtype GPR84-eYFP and each of the Ser^221^Ala, Ser^224^Ala-GPR84 and Thr^263^Ala, The^264^Ala-GPR84-eYFP mutants were transiently expressed for arrestin-3 (*D*) or arrestin-2 (*E*) proximity assays in response to varying concentrations of 2-HTP. The same receptor constructs as in *D* and *E* but now lacking the C-terminal eYFP tag were expressed transiently and used in arrestin-3 bystander BRET assays (*F*). The same eYFP-containing constructs as in *D* and *E* but now expressed stably in Flp-In TREx 293 cells were used to measure 2-HTP-mediated regulation of cAMP levels (*G*). One-way ANOVA followed by Dunnett's multiple comparisons test. Data are means ± SEM. n ≥ 3.

Although recognized effectively by the GPR84 Ct-structural antiserum (Fig. 7*A*), unsurprisingly, the anti-pSer^221^/pSer^224^ antiserum was unable to recognize pmIL3-hGPR84-eYFP as these serines are replaced by alanines (Fig. 7*B*). This was also the case for the anti-pThr^263^/pThr^264^ antiserum (Fig. 7*C*). Although the construct was not expressed to the same level as hGPR84-eYFP, or indeed pmIL3-hGPR84-eYFP (Fig. 7*A*), replacement of only Ser^221^ and Ser^224^ by alanines prevented recognition by the anti-pSer^221^/pSer^224^ antiserum (Fig. 7*B*) and equivalent replacement of Thr^263^ and Thr^264^ by alanines prevented recognition of this variant by anti-pThr^263^/pThr^264^ (Fig. 7*C*) confirming the specificity of these antisera. In 2-HTP-induced arrestin-3 interaction studies, although alteration of Ser^221^ and Ser^224^ had no effect on either agonist potency or the magnitude of effect, mutation of Thr^263^and Thr^264^ resulted in a form of the receptor that was coupled only weakly (*p* < 0.0001) to arrestin-3 interaction in response to 2-HTP, and with significantly reduced potency (*p* < 0.001) (Fig. 7*D*). Outcomes using this mutant were akin to the pmIL3-hGPR84-eYFP mutant in which all the Ser-Thr residues within IL3 were replaced, suggesting that phosphorylation of this pair of Thr residues provides much of the affinity that supports GPR84-arrestin-3 interactions. As interactions of GPCRs with the two ubiquitously expressed arrestin isoforms arrestin-2 and arrestin-3 can be different (31, 32), we repeated these experiments using instead arrestin-2-nanoluciferase. Similar to the arrestin-3 studies, alteration of Ser^221^ and Ser^224^ in GPR84 had no effect on either agonist potency or the magnitude of effect (Fig. 7*E*), whereas interactions and/or proximity with arrestin-2 measured in these assays after mutation in GPR84 of Thr^263^and Thr^264^ to alanine were virtually undetectable following addition of 2-HTP (*p* < 0.0001) (Fig. 7*E*). As these assays simply measure induced proximity between the eYFP of the tagged receptor and the nanoluciferase in the arrestin constructs we expanded these studies using so-called bystander BRET studies (33, 34) in which the transfected receptor is untagged. Such studies confirmed the poor interaction of Thr^263^Ala,Thr^264^Ala-GPR84 with arrestin-3 (Fig. 7*F*). By contrast, this mutant did not alter G protein–mediated signaling as assessed in cAMP regulation assays in response to 2-HTP (Fig. 7*G*).

**Figure 8:**
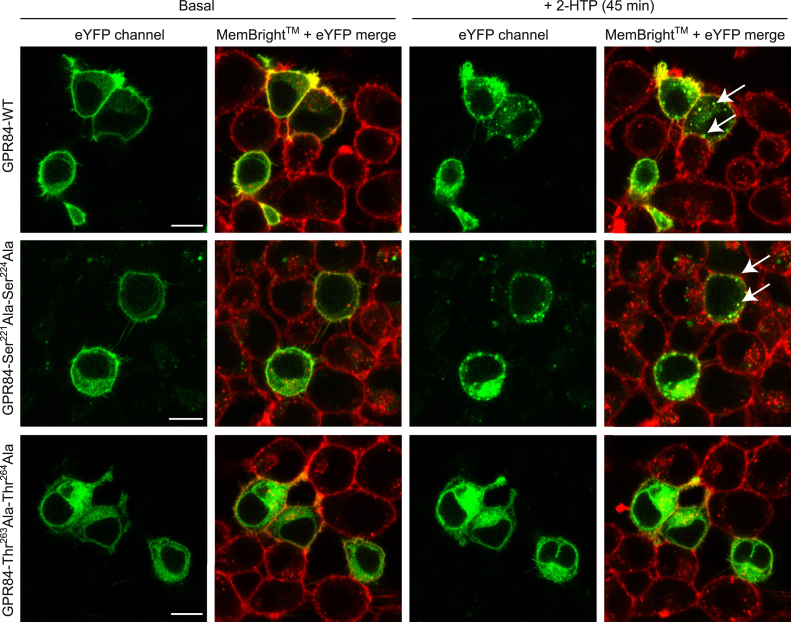
**Thr**^**263**^**and Thr**^**264**^**are required for agonist-induced internalization of GPR84,** Wildtype-, Ser^221^Ala,Ser^224^Ala-, and Thr^263^Ala,Thr^264^Ala-GPR84-eYFP (*green*) were transiently expressed in HEK293T cells and exposed to 2-HTP (10 μM) for 0 (basal) or 45 min. Subsequently cells were labeled with Lipilight MemBright 640 (*red*) and imaged. 2-HTP promoted internalization of wildtype- and Ser^221^Ala,Ser^224^Ala-GPR84-eYFP (*illustrative arrows*) but not Thr^263^Ala,Thr^264^Ala-GPR84-eYFP. The scale bar represents 10 μm. Representative images are shown.

As effective interactions with arrestin isoforms are often associated with agonist-induced GPCR internalization we next assessed whether these phosphorylation-site mutants might be affected following transient introduction of each of wildtype-, Ser^221^Ala, Ser^224^ Ala-, and Thr^263^Ala,Thr^264^Ala-GPR84-eYFP into HEK293 cells. After treatment with 2-HTP for 45 min the cellular location of the receptor constructs was compared with the basal state, with MemBright-640 dye used to identify the cell surface membrane, including in cells that did not express the receptor constructs (Fig. 8). Although a significant amount of each construct was located intracellularly in the basal state, something that may relate directly to the apparent immature forms detected in the immunoblot studies (Figs. 5 and 6), 2-HTP promoted internalization into punctate vesicles of wildtype- and Ser^221^Ala, Ser^224^ Ala-GPR84-eYFP (Fig. 8) but was without a detectable effect on Thr^263^Ala,Thr^264^Ala-GPR84-eYFP (Fig. 8).

**Figure 9:**
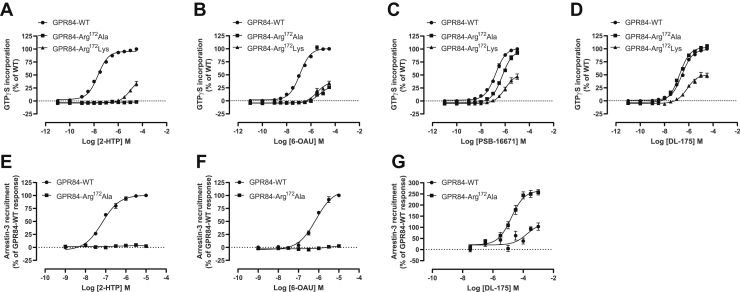
**DL-175 does not require Arg**^**172**^**for function at GPR84**. The ability of various concentrations of 2-HTP (*A*), 6-OAU (*B*), PSB-16671 (*C*), and DL-175 (*D*) to stimulate binding of [^35^S]GTPγS was assessed in membranes of Flp-In TREx 293 induced to express wildtype, Arg^172^Ala, or Arg^172^Lys human GPR84-Gα_i2_ fusion proteins. The ability of various concentrations of 2-HTP (*E*), 6-OAU (*F*), and DL-175 (*G*) to stimulate arrestin-3 recruitment was assessed in cells transfected with either wildtype or Arg^172^Ala GPR84-eYFP. Data are means ± SEM. n ≥ 3.

**Figure 10:**
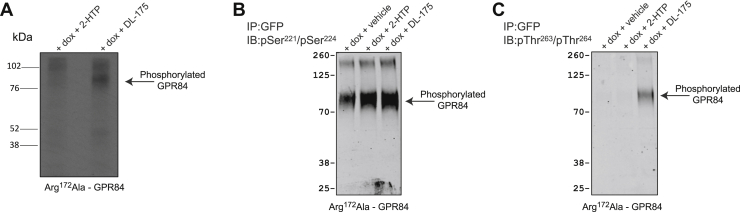
**DL-175 promotes phosphorylation of residues Thr**^**263**^**/Thr**^**264**^**in Arg**^**172**^**Ala GPR84.** Phosphorylation of Arg^172^Ala GPR84 was measured in Flp-In TREx 293 induced (+dox) to express this receptor upon addition of 2-HTP or DL-175 (*A*). Although DL-175 did not affect the constitutive phosphorylation of Ser^221^/Ser^224^ (*B*), it did promote phosphorylation of residues Thr^263^/Thr^264^ (*C*). Representative experiments are displayed. dox, doxycycline.

To attempt to consider the variation in effects of the different GPR84 ligands across these studies we attempted to define their modes of binding. We have previously predicted a key role in orthosteric agonist recognition for Arg^172^, located with the second extracellular loop (EL2) of GPR84, which in homology models we have predicted to point inward into the orthosteric binding cavity (24, 35). Because effects of MCFAs at GPR84 are lacking at an Arg^172^Ala GPR84 mutant (24) we have suggested a key role for the positive change of Arg^172^ to coordinate the aliphatic carboxylate of MCFAs. Endogenous activators of GPCRs are defined as being orthosteric agonists of their corresponding receptor. Both 2-HTP and 6-OAU can therefore also be defined as orthosteric agonists of GPR84 in that their function was also lacking when assessed at a Arg^172^Ala GPR84 mutant (Fig. 9, *A* and *B*). By contrast, function of the allosteric agonist PSB-16671 was little affected at this mutant with only a small reduction in ligand potency and no decline in efficacy (Fig. 9*C*). Because DL-175 was developed based on predicted similarities to 6-OAU (21), it was initially surprising that the potency and efficacy of DL-175 was maintained at Arg^172^Ala GPR84 (Fig. 9*D*). Further mutagenesis to generate Arg^172^Lys GPR84 showed that function of both 2-HTP and 6-OAU was again lacking at this mutant in [^35^S]GTPγS binding assays (Fig. 9, *A* and *B*). Although both PSB-16671 and DL-175 were still active at Arg^172^Lys GPR84, they both displayed reduced potency and efficacy at this mutant (Fig. 9, *C* and *D*). Of interest, however, in arrestin-3 interaction studies, although 2-HTP and 6-OAU again lacked function at Arg^172^Ala GPR84 (Fig. 9, *E* and *F*), now DL-175 displayed gain of function compared with its effect at the wildtype receptor (Fig. 9*G*). Consistent with this observation, DL-175 was able to promote incorporation of [^32^P] into the mutated Arg^172^Ala GPR84 receptor after labeling of cells with [^32^P] orthophosphate (Fig. 10*A*), whereas, as predicted from other studies, 2-HTP was not (Fig. 10*A*). Moreover, although Arg^172^Ala GPR84 was, like wildtype GPR84, constitutively phosphorylated at Ser^221^/Ser^224^ and this was unaffected by treatment with DL-175 (Fig. 10*B*), the anti-pThr^263^/pThr^264^ antiserum indicated that at this mutant these residues also became phosphorylated in response to DL-175 (Fig. 10*C*), consistent with these sites and their phosphorylation status providing a biosensor for arrestin isoform engagement.

**Figure 11:**
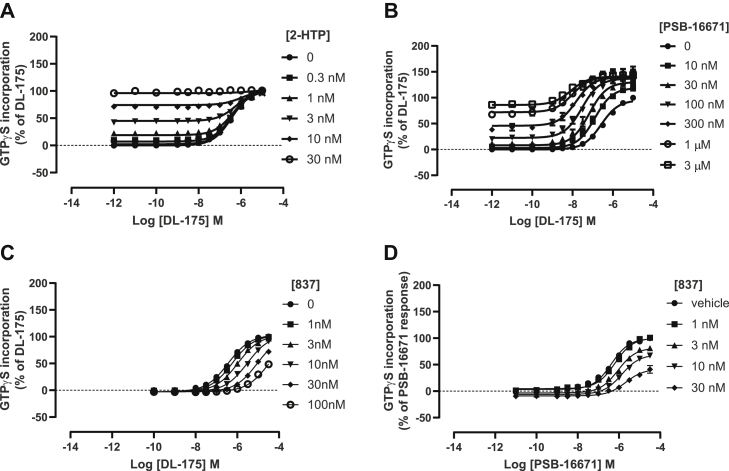
**DL-175 acts as an orthosteric agonist of GPR84.** Similar experiments to those of Figure 9 using GPR84-Gα_i2_ expressing membranes assessed how the potency or DL-175 might be modulated in the presence of increasing concentrations of 2-HTP (*A*) or PSB-16671 (*B*). Increasing concentrations of antagonist 837 altered the observed potency of DL-175 in a manner consistent with the two ligands binding competitively. Schild slope = 1.04 ± 0.05, pA2 compound 837 = 8.84 ± 0.06 (*C*). By contrast, the effect of antagonist 837 on the function of PSB-16671 was consistent with noncompetitive interactions (*D*). Data are means ± SEM. n ≥ 3.

Although DL-175 and 2-HTP displayed different functionality at the Arg^172^Ala GPR84 mutant, coaddition studies using the wildtype receptor indicated that they must overlap in their sites of binding. This could be concluded because increasing concentrations of 2-HTP did not alter the observed EC_50_ of DL-175 in [^35^S]GTPγS binding assays (Fig. 11*A*). By contrast, as we have previously shown for 2-HTP [**7**], the observed EC_50_ for DL-175 was increased by the presence of the allosteric agonist PSB-16671 (Fig. 11*B*), with calculated effects reflecting both increased affinity and efficacy of DL-175. This is consistent with DL-175 and PSB-16671 binding to topographically distinct sites. Additional support for the concept that DL-175 binds and acts in an orthosteric manner was that the effect of DL-175 in [^35^S]GTPγS binding assays was shifted to higher concentrations, but in a manner that could be fully overcome, by increasing concentrations of the competitive orthosteric GPR84 antagonist compound 837 (11) (Fig. 11*C*). By contrast, the effect of PSB-16671, although blocked by increasing concentrations of compound 837, this effect of the antagonist was clearly produced in a noncompetitive manner as increasing concentrations of compound 837 depressed the maximal response to PSB-16671 (Fig. 11*D*).

**Figure 12:**
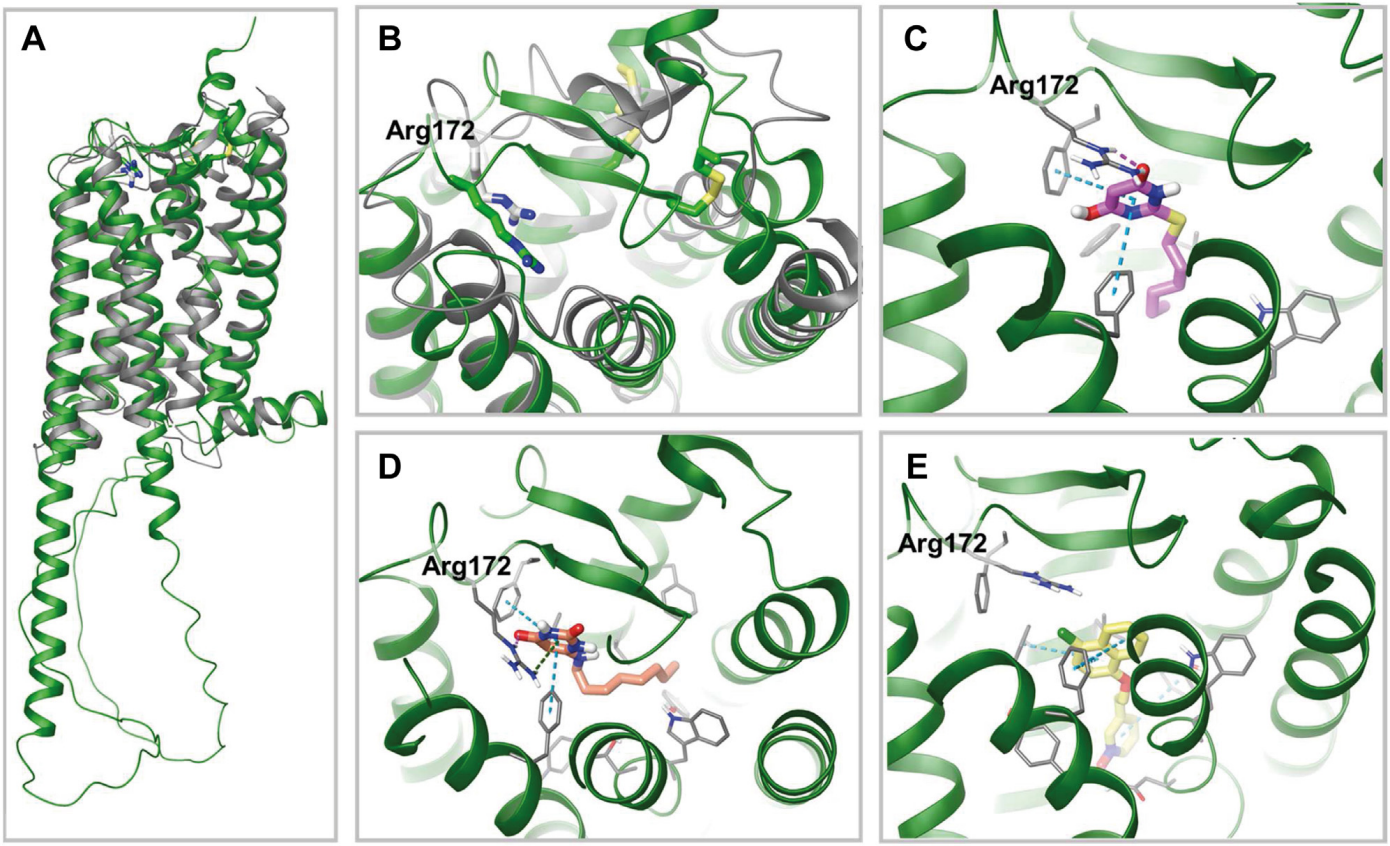
**3D computational models of GPR84 and predicted interactions with agonists.***A*, the overlay of the hybrid template homology model (in *gray*) and “AlphaFold” structure (in *green*) of GPR84. *B*, the view of overlay from the extracellular side with visualized Arg^172^ and disulfide bridges. Both models predict the second extracellular loop as a β-sheet and Arg^172^ pointed into the extracellular opening of the helical bundle. The binding mode of 2-HTP (*C*) (in *pink*), 6-OAU (*D*) (in *orange*), and DL-175 (*E*) (in *yellow*) in the AlphaFold model. Hydrogen bonds, π–π, and cation–π interactions are shown in a *pink*, *cyan*, and *green dashed line*.

We have previously developed and employed hybrid template homology models of GPR84 that predict the positively charged guanidinium head group of Arg^172^ pointed into the extracellular opening of the receptor helical bundle (11, 35). This is distinct from other models of GPR84 developed (36). The comparison of the hybrid template homology model with the GPR84 structure generated by “AlphaFold” (37) (https://alphafold.ebi.ac.uk/entry/Q9NQS5) shows high similarity (Fig. 12, *A* and *B*). We therefore used the AlphaFold template to dock 2-HTP (Fig. 12*C*), 6-OAU (Fig. 12*D*), and DL-175 (Fig. 12*E*). From docking, both 2-HTP and 6-OAU interact with Arg^172^, whereas DL-175 sits deeper in the intrahelical cavity without direct contact with Arg^172^. The interaction of the carboxylic acid bioisosteres of 2-HTP and 6-OAU with Arg^172^ is similar to a salt-bridge interaction known for other receptors binding free fatty acids (38) and is in accord with the loss of function of these ligands with mutational removal of Arg^172^. DL-175 does not possess an equivalent polar group. Thus, Arg^172^ is not critical for binding of DL-175, as supported by mutagenesis.

## Discussion

GPR84 is attracting considerable interest as a potential therapeutic target because of its pattern of expression, largely by immune cell types, and the substantial upregulation in both mRNA and protein amounts in proinflammatory settings. Indeed, the best characterized high-affinity GPR84 antagonist, GLPG1205, has been, and is being, assessed clinically in both ulcerative colitis and idiopathic pulmonary fibrosis (8). Although there is a general paucity of high-affinity GPR84 antagonists reported, a substantial number of ligands have been shown to have agonist function. These include 2-HTP, 6-OAU, and embelin (1, 3). Recently DL-175 was introduced as a GPR84 agonist that shows markedly different signaling characteristics compared with 6-OAU (21) with DL-175 reported to be unable to promote interactions between GPR84 and arrestin adaptor proteins while being an effective modulator of G protein signaling and hence able to reduce cellular levels of cAMP. It is hence a functionally biased ligand. We confirmed that DL-175 is unable to promote effective engagement with arrestin-3 *via* wildtype GPR84, whereas both 2-HTP and 6-OAU do so. Because effective engagement with arrestins is frequently dependent on ligand-induced phosphorylation of various Ser and Thr residues in either or both the Ct and IL3 of receptors we explored this in detail. We adopted a range of approaches to do so. Initially, direct incorporation of [^32^P] into the receptor was assessed both in the absence and presence of 2-HTP, and we subsequently assessed whether 6-OAU, DL-175, and PSB-16771 were able to do likewise. We then determined whether sites of such phosphorylation induced by 2-HTP were located in the IL3, the Ct, or both and observed that mutation to Ala of all 21 potential sites within IL3 completely prevented phosphorylation. As this was too large a number of sites to investigate in a systematic and sequential manner by targeted mutagenesis, we turned to analysis by mass spectrometry. Two key outcomes from these studies were that both Ser^221^ and Ser^224^ were constitutively phosphorylated in the absence of 2-HTP, whereas a number of other residues were only detected as being phosphorylated after treatment of cells with 2-HTP, indicating these to be dynamically regulated.

Based on the patterns of phosphorylation observed we generated antisera designed to selectively identify phosphorylated residues in hGPR84. Of these the pSer^221^/pSer^224^ antiserum identified the receptor both in the absence and presence of prestimulation with 2-HTP. These sites were clearly phosphorylated because recognition by this antiserum was lost when samples were pretreated with λ-PPase that removes phosphate groups from proteins. By contrast, pThr^263^/pThr^264^ antisera only recognized hGPR84 in immunoblotting studies following prior cell treatment with 2-HTP, and once again studies with λ-PPase confirmed protein recognition did indeed reflect protein phosphorylation. It is well appreciated that agonist-induced phosphorylation often increases the affinity of interaction of a receptor and arrestin. However, it is rarely clear exactly which Ser/Thr residues are key to this process. Of note, mutation to Ala of only Thr^263^and Thr^264^ produced a form of hGPR84 that was as poor in 2-HTP-mediated arrestin-2 and arrestin-3 interactions as the mutant in which we altered to Ala all 21 Ser/Thr in IL3. This indicates Thr^263^and Thr^264^ play a key role. Given the frequently dominant role of GRK-mediated phosphorylation in driving enhanced-arrestin interactions with receptors it was perhaps not surprising that 2-HTP-mediated phosphorylation of Thr^263^/Thr^264^ was prevented by GRK2/3 inhibition. However, not all phosphorylation of GPR84 is produced in an agonist-regulated manner. Ser^221^/Ser^224^ were phosphorylated constitutively, and these posttranslational modifications also did not contribute substantially to agonist-driven interactions with arrestin-3. A major challenge in understanding such roles is that there are no current atomic level structures of GPR84. Moreover, with a long internal IL3 it is unlikely that any structural aspects of this would be resolved, even if this sequence was not eliminated in efforts to produce a more homogenous protein source for crystallization trials. However, it is noticeable that, in the recently released “AlphaFold” structural prediction for human GPR84 (https://alphafold.ebi.ac.uk/entry/Q9NQS5) (37), an alpha helix is predicted to extend substantially into IL3 after transmembrane domain 5 (see Fig. 12*A*) and Ser^221^ and Ser^224^ are predicted to be part of this alpha helix. This is likely why these residues are three amino acids apart in the primary sequence as they would be expected to neighbor one another on the same face of the helix.

To try to gain insight into why DL-175 acted very differently from 2-HTP and 6-OAU in terms of promoting GPR84 phosphorylation of Thr^263^/Thr^264^ and hence arrestin-3 interactions we first considered whether DL-175 is actually an orthosteric agonist. Despite not losing function at an Arg^172^Ala mutation of GPR84 that eliminates the function of other orthosteric agonists including 2-HTP and 6-OAU, and indeed MCFAs that are the potential endogenous regulators of GPR84 (24), DL-175 was indeed orthosteric. For example, the orthosteric antagonist compound 837 (11) was fully competitive with DL-175, whereas PSB-16671 acted as a positive allosteric modulator of DL-175, as it does with other orthosteric agonists, including 2-HTP. Based on homology modeling we have suggested that MCFAs and 2-HTP make a key interaction involving Arg^172^, with the side-chain guanidinium of this Arg pointing inward from its backbone location in EL2 of the receptor (24, 35). Such models are helpful but require further support and validation. As noted earlier, although an atomic level structure of GPR84 is not currently available, the structural prediction in the AlphaFold database is entirely consistent with our models. AlphaFold is a deep learning algorithm that combines physics, biology, and evolution of proteins from the Protein Data Bank and multisequence alignments to predict the unknown protein structure (37). AlphaFold has been demonstrated to be able to predict protein structures to almost experimental accuracy in the 14th Critical Assessment of protein Structure Prediction (CASP14) (39). It is anticipated that in the case of GPCRs fold predictions the performance of AlphaFold should be trustworthy since a number of experimental GPCRs structures were available to be used as a starting point in the training of the neural network. However, predictions of “active” and “inactive” receptor conformations using AlphaFold are still challenging. The AlphaFold per-residue confidence score for the putative orthosteric site and EL2 of the AlphaFold GPR84 model is at “very high” or “confident” (for Arg^172^) levels. We, therefore, used the AlphaFold model together with our previous homology model for docking of 2-HTP, 6-OAU, and DL-175 to provide insight into agonist binding.

In conclusion, we demonstrate that sites of both constitutive and agonist-regulated phosphorylation are present within IL3 of human GPR84. Remarkably, given the number of serine and threonine residues region, the phosphorylation of Thr^263^ and Thr^264^ provides much of the affinity required for interactions with arrestin-3. The production of a set of antisera that specifically identified these residues when phosphorylated offers biosensors to assess the extent of GPR84 activation *in situ*. As GPR84 is markedly upregulated in proinflammatory settings these may be useful in assessing the degree of activation of the receptor in these settings as well as increased levels of the receptor. In addition, the homology model of GPR84 that we had developed independently overlays exceptionally well with the AlphaFold structural prediction, and these provided strong rationale for the location and directionality of Arg^172^ in the EL2, which we have previously predicted to coordinate the carboxylate head-group of MCFAs and the carboxylate bioisosteres present in many synthetic GPR84 orthosteric agonists but not in the biased agonist DL-175.

## Experimental procedures

### Materials

6-n-Octylaminouracil (6-OAU) and 2-(hexylthio)-6-hydroxy-4(3H)-pyrimidinone (2-HTP) were from Sigma-Aldrich. (3-((5,6-Bis(4-methoxyphenyl)-1,2,4-triazin-3-yl)methyl)-1*H*-indole) (837) was synthesized as in Jenkins *et al*., [**ref 11**]. 9-Cyclopropylethynyl-2-((*S*)-1-[1,4]dioxan-2-ylmethoxy)-6,7-dihydropyrimido[6,1-*a*]isoquinolin-4-one (GLPG1205) and di(5,7-difluoro-1H-indole-3-yl)methane (PSB-16671) were kindly provided by Galapagos NV. [^35^S]GTPγS was from PerkinElmer Life Sciences. Tissue culture reagents, NuPAGE Novex 4% to 12% Bis-Tris Gels and NuPAGE MOPS SDS running buffer were from Thermo Fisher Scientific, and molecular biology enzymes, reagents, and Nano-Glo Luciferase assay substrate were from Promega. Polyethylenimine (PEI) (linear poly(vinyl alcohol) [MW 25,000]) was from Polysciences. Lambda protein phosphatase (λ-PPase) was from New England BioLabs. 3-(2-((4-Chloronaphthalen-1-yl)oxy)ethyl)pyridine 1-oxide (DL-175) and compound 101 were from Tocris Bioscience. Complete protease inhibitors mixture and phosphatase inhibitor tablets were from Roche Diagnostics. Lipilight MemBright 640 was from Idylle.

### Antibodies

The rabbit phospho-site-specific GPR84 antibodies pSer^221^/pSer^224^-GPR84 (7TM0120A) and pThr^263^/pThr^264^-GPR84 (7TM0120B) were developed in collaboration with 7TMAntibodies GmbH. The structural anti-GPR84 antibody, which detects GPR84 in a phosphorylation-independent manner was from 7TMAntibodies (7TM0120N). IRDye 800CW donkey anti-rabbit IgG was from LI-COR Biosciences. Anti-GFP antisera were from Abcam (ab1218) or in-house generated sheep anti-GFP.

### Generation of constructs

FLAG-human GPR84-eYFP and FLAG-human GPR84-Gα_i2_ fusion proteins were constructed as described (7, 24). mNeonGreen fused to the fatty acylation motif of Lyn-kinase was subcloned after PCR amplification (using primers designed to add NheI and EcoR1 sites) into the NheI and EcoR1 site in the MCS1 of pIRES vector. Subsequently, arrestin-3-nanoluciferase was subcloned after PCR amplification (using primers designed to add Xba1 and NotI sites) into the Xba1 and NotI sites in the MCSII of the above vector.

### Mutagenesis of FLAG-human GPR84-eYFP and FLAG-human GPR84-Gα_i2_

The Stratagene QuikChange method (Stratagene, Agilent Technologies) was used to introduce alterations into FLAG-human GPR84-eYFP or FLAG-human GPR84-Gα_i2_. Primers utilized for mutagenesis were provided by MWG Operon. Template DNA was digested with DpnI to leave only the newly synthesized mutated plasmid, and sequencing was carried out to confirm the introduction of the alterations.

### Cell culture, transfection, and generation of cell lines

HEK293T cells were maintained in Dulbecco’s modified Eagle’s medium supplemented with 0.292 g/l L-glutamine, 1% penicillin/streptomycin mixture, and 10% heat-inactivated fetal bovine serum at 37 °C in a 5% CO_2_ humidified atmosphere. HEK293T cells were transfected using PEI. The day before transfection 2 × 10^6^ cells were plated into 10-cm dishes. Plasmid DNA was then combined with PEI (in 1:6 ratio) in 500 μl of 150 mM NaCl, thoroughly mixed and incubated for 10 min at room temperature. Cell medium was changed, and the DNA–PEI mixture was added to the medium in a dropwise manner.

Flp-In TREx 293 cells (Invitrogen) were maintained in Dulbecco's modified Eagle's medium without sodium pyruvate, supplemented with 10% (v/v) fetal bovine serum, 1% penicillin/streptomycin mixture, and 10 μg/ml blasticidin at 37 °C in a 5% CO_2_ humidified atmosphere. To generate Flp-In TREx 293 cells able to express in an inducible manner the various GPR84 receptor constructs, cells were transfected with a mixture containing the desired cDNA in pcDNA5/FRT/TO vector and pOG44 vector (1:9) by using 1 mg/ml PEI (MW 25,000). Cells were plated until 60% to 80% confluent then transfected with 8 μg of required plasmid DNA and PEI (ratio 1:6 DNA/PEI), diluted in 150 mM NaCl, pH 7.4. After incubation at room temperature for 10 min, the mixture was added to cells. After 48 h, the medium was changed to medium supplemented with 200 μg/ml hygromycin B to initiate the selection of stably transfected cells. After isolation of resistant cells, expression of the appropriate construct from the Flp-In TREx locus was induced by treatment with up to 100 ng/ml doxycycline for 24 h.

CHO-K1 cells stably expressing hGPR84-eYFP receptor were maintained in Ham's F-12 medium supplemented with 10% fetal calf serum, penicillin (50 units/ml), streptomycin (50 μg/ml), and geneticin G418 (500 μg/ml).

### [^35^S]GTPγS incorporation assay

Prepared membrane protein (3 μg Flp-In T-REx 293 cells) was incubated in assay buffer (20 mM Hepes, 5 mM MgCl_2_, 160 mM NaCl, 0.05% fatty-acid-free bovine serum albumin; pH 7.5) containing the indicated ligand concentrations. The reaction was initiated by addition of [^35^S]GTPγS (50 nCi per reaction) with 1 μM GDP, and incubated at 30 °C for 60 min. The reaction was terminated by rapid vacuum filtration through GF/C glass fiber filter-bottom 96-well microplates (PerkinElmer Life Sciences) using a UniFilter FilterMate Harvester (PerkinElmer). Unbound radioligand was removed from filters by three washes with ice-cold PBS. MicroScint-20 (PerkinElmer) was added to dried filters, and [^35^S]GTPγS binding was quantified by liquid scintillation spectroscopy.

### HTRF-based cAMP inhibition assays

cAMP experiments were performed using Flp-In T-REx293 cells induced to express the receptor construct of interest. Experiments were carried out using a homogenous time-resolved FRET-based detection kit (CisBio) according to the manufacturer’s protocol. For the assay cells were plated at 5000 cells/well in low-volume 384-well plates. The ability of agonists to inhibit 1 μM forskolin-induced cAMP production was assessed following 1 h incubation with agonist compounds. Reactions were stopped according to the manufacturer’s instructions, and the output was measured with a PHERAstar FS plate reader (BMG Labtech).

### Bioluminescence resonance energy transfer–based arrestin-2 and arrestin-3 recruitment assays

HEK293T cells were transiently transfected with human wildtype or each of the indicated GPR84 mutants C-terminally tagged with eYFP and either arrestin-2 or arrestin-3 fused to nanoluciferase in a ratio of 100:1, respectively. Transfection with the appropriate arrestin isoform fused to nanoluciferase alone was performed as control. After 24 h, cells were seeded at 50,000 cells per well in poly-D-lysine-coated 96-well plates and incubated at 37 °C overnight. After 24 h, cells were washed twice with Hank’s balanced salt solution (HBSS), pH 7.4, and 10 μl nano-Glo luciferase substrate, diluted 1:80, was added to each well. Cells were incubated in the dark for 10 min at 37 °C. Following agonist compound addition, cells were incubated for further 5 min at 37 °C before sequential reading of emission signals on a PHERAstar FS plate reader (BMG Labtech) at 475 and 535 nm, representing nanoluc luciferase and eYFP emission signals, respectively. The net bioluminescence resonance energy transfer (BRET) ratio (mBRET) was calculated as follows: [(signal 535 nm/signal 475 nm) - (signal nanoluc luciferase only 535 nm/signal nanoluc luciferase only 475 nm)] ∗1000.

### GPR84-arrestin bystander BRET assays

HEK293T cells were cotransfected with cDNA encoding both the fluorescent protein mNeonGreen fused to the fatty acylation motif of Lyn-kinase and arrestin-3-nanoluciferase, along with wildtype or mutated forms of GPR84. Post transfection cells were cultured at 37 °C in a 5% CO_2_ humidified atmosphere for 24 h, transferred to poly-D-lysine-precoated 96-well plates at a density of 5 × 10^4^ cells per well, and maintained as above for a further 16-h period. Cells were then washed once with HBSS and incubated in 80 μl HBSS for 30 min at 37 °C. A volume of 10 μl nano-Glo luciferase substrate, diluted 1:80, was added to each well, and cells were incubated in the dark for 10 min at 37 °C. Dual 535- and 475-nm luminescent emission measurements were recorded using a PherStar FS plate reader (BMG Labtech) at 1.5-min intervals for 6 min prior to and 39 min following the addition of the agonist compound 2-HTP. Net BRET was calculated as the 535 nm/475 nm ratio after correcting for both the well baseline and test compound vehicle response. BRET data were reported by plotting the Net BRET value at 1080 s *versus* the concentration of 2-HTP.

### Plasma membrane imaging and receptor internalization

HEK293T cells were seeded at 0.5 x 10^5^ cells/well on poly-D-lysine-coated 30-mm round coverslips in 6-well plates and incubated for 24 h at 37 °C. Cells were transiently transfected with wildtype or GPR84 mutants each C-terminally tagged with eYFP. After 48 h, cells were washed twice with HBSS and incubated with 100 nM MemBright 640 solution for 10 min at 37 °C. Subsequently, coverslips were placed in a microscope chamber containing HBSS and images were acquired before treatment and 45 min after agonist addition using a Zeiss LSM 880 confocal equipped with a 63x/1.4 NA Plan Apochromat oil-immersion objective. MemBright 640 dye was excited using 633-nm laser light, and emission light was detected over the wavelength range 660 to 700 nm.

### Detection of ligand-dependent phosphorylation using [^32^P] orthophosphate

Cells were plated onto poly-D-lysine-coated 6-well plates at 4 × 10^5^ cells per well. These were allowed to grow overnight and were then induced with 100 ng/ml doxycycline. After 24 h the media were removed and the cells washed with 3 × 2 ml per well phosphate-free Krebs solution (10 mM Hepes, 118 mM NaCl, 1.3 mM CaCl_2_•2H_2_O, 4.3 mM KCl, 1.17 mM MgSO_4_•7H_2_O, 4.17 mM NaHCO_3_, and 11.7 mM glucose, pH 7.4). After removal of the last wash, 1 ml Krebs solution containing 100 μCi [^32^P] orthophosphate was added per well and the plate incubated at 37 °C for 1 h. Ligand and other treatments were carried out as described specifically in the text, but generally antagonists were applied 45 min after the addition of Krebs solution containing [^32^P] orthophosphate (−15 min) and agonists after 55 min (−5 min).

The plates were put on ice and the medium was removed, 1 ml RIPA buffer (10 mM Tris-HCl, 2 mM EDTA, 20 mM glycerol-2-phosphate, 160 mM NaCl, 1% (v/v) Nonidet-P40, and 0.5% (w/v) sodium deoxycholate pH 7.4, supplemented with protease and phosphatase inhibitor tablets) was added per well, and the cells were scraped using the wide end of a 1-ml pipette tip before being transferred to precooled Eppendorf tubes. The cell suspension was pipetted up and down 20 times to lyse the cells and then spun at 20,000*g* for 10 min at 4 °C. A volume of 900 μl of supernatant was then transferred to precooled Eppendorf tubes. Anti-GFP antibody, 1 μl, and 200 μl protein-A Sepharose (6% v/v from a 50% slurry of beads in TEG buffer [10 mM Tris-HCl, 2 mM EDTA and 0.1% glycerol pH 7.4 supplemented with protease and phosphatase inhibitor tablets]) were added and the tubes incubated on a wheel at 4 °C overnight.

The tubes were spun at 200*g* for 1 min at 4 °C, and the supernatant was removed and discarded. The beads were washed with 3 × 500 μl TEG buffer (spinning as above between washes) and as much of the TEG removed as possible. A volume of 30 μl 2 × SDS-PAGE sample buffer (125 mM Tris-HCl, 200 mM dithiothreitol, 4% (w/v) SDS, 20% (v/v) glycerol, and 0.05% (w/v) bromophenol blue, pH 6.8) was added, and the tubes were heated to 55 to 60 °C for 5 min and then spun at 20,000*g* for 5 min at 4 °C. The samples (20 μl) were resolved on SDS-PAGE, and the gels were dried before exposure to film at −80 °C in a cassette with intensifying screens.

In order to assess the amounts of protein loaded, further SDS-PAGE gels were run with the remaining sample and the proteins transferred to nitrocellulose, which was then blocked (5% fat-free milk powder in phosphate buffered saline [PBS], 120 mM NaCl, 25 mM KCl, 10 mM Na_2_HPO_4_, and 3 mM KH_2_PO_4_, pH7.4, with 0.1% Tween-20 [PBS-Tween]) at 4 °C on a rotating shaker overnight. The membrane was incubated for 3 h with primary antibody (1:10,000 sheep anti-GFP) in 2% fat-free milk powder in PBS-Tween, washed (3 × 10 min PBS-Tween), and then incubated for 3 h with appropriate secondary antibody (horseradish peroxidase–linked rabbit anti-goat IgG, diluted 1:10,000 in 2% fat-free milk powder in PBS-Tween). After washing as above, signal was detected by enhanced chemiluminescence (Pierce Chemical) according to the manufacturer’s instructions.

### Cell lysate preparation

Cell lysates were generated from Flp-In TREx 293 cells following 100 ng/ml doxycycline treatment to induce eYFP- fusion receptor expression. Cells were harvested in ice-cold PBS and lysed in lysis buffer (150 mM NaCl, 50 mM Tris-HCl, 5 mM EDTA, 1% Nonidet P-40, 0.5% Na-deoxycholate, and 0.1% SDS, supplemented with complete protease inhibitors mixture and phosphatase inhibitor tablets) on a rotating wheel for 30 min at 4 °C. Samples were then centrifuged for 15 min at 21,000*g* at 4 °C. The protein content was assessed using a BCA protein assay kit (Thermo Fisher Scientific).

### Receptor immunoprecipitation and immunoblotting assays

eYFP-linked receptor constructs were immunoprecipitated from 200 μl cell lysate (5 μg/μl of protein) using the GFP-Trap kit (Chromotek) according to manufacturer's instructions. Immune complexes were washed three times in washing buffer, resuspended in 100 μl 2 × SDS-PAGE sample buffer and incubated at 60 °C for 10 min. Following centrifugation at 2500*g* for 5 min, 20 μl of immunoprecipitated proteins was resolved by SDS-PAGE on 4% to 12% BisTris (3) gels. After separation, the proteins were transferred electrophoretically onto nitrocellulose membrane, which was then blocked using 5% bovine serum albumin (BSA) in Tris-buffered saline (TBS, 50 mM Tris-Cl, 150 mM NaCl, pH 7.6) for 1 h at room temperature on a rotating shaker. The membrane was then incubated with appropriate primary antibody in 5% BSA powder in TBS supplemented with 0.1% Tween (TBS Tween) overnight at 4 °C on a rotating shaker. Anti-GPR84, anti-pSer^221^/pSer^224^, and anti-pThr^263^/pTh^r264^ antisera were diluted 1:2000. Subsequently, the membrane was washed (3 × 10 min with TBS-Tween) and incubated for 2 h with anti-rabbit secondary antibody diluted 1:10,000 in 5% BSA in TBS-Tween. After washing (3 × 10 min with TBS-Tween), proteins were detected using Odyssey imaging system according to the manufacturer's instructions.

#### Cell treatment with compound 101

to inhibit GRK2/3 function, cells were treated with 10 μM compound 101 for 30 min at 37 °C in a 5% CO_2_ humidified atmosphere, prior to ligand treatment.

### Cell lysate treatment

To remove phosphate groups, immunocomplexes were treated with λ-PPase at a final concentration of 10 unit/μl for 90 min at 30 °C before elution with 2 × SDS-PAGE sample buffer as described earlier.

### Membrane preparation

Membranes were generated from Flp-In T-REx 293 cells following 100 ng/ml doxycycline treatment to induce receptor expression. Cells were washed with ice-cold PBS, removed from dishes by scraping, and centrifuged at 1200 rpm for 5 min at 4 °C. Pellets were resuspended in TE buffer (10 mM Tris-HCl, 0.1 mM EDTA pH 7.5) containing a protease inhibitor mixture and homogenized with a 5-ml hand-held homogenizer. This material was centrifuged at 1500 rpm for 5 min at 4 °C, and the supernatant was further centrifuged at 50,000 rpm for 45 min at 4 °C. The resulting pellet was resuspended in TE buffer, and the protein content was assessed using a BCA protein assay kit.

### Mass spectrometry

Flp-In T-REx 293 cells harboring GPR84-eYFP or CHO-K1 cells stably expressing this construct were used. Mass spectrometry was performed at the University of Leicester Proteomics Facility. Samples were analyzed as described (40). Liquid chromatography/tandem mass spectrometry (LC-MS/MS) was carried out using an LTQ Orbitrap mass spectrometer (Thermo Fisher Scientific). A reverse-phase trapping column (0.3 mm inner diameter x 1 mm) containing 5 μm C18 300 Å Acclaim PepMap medium (Dionex) was loaded with the tryptic peptides at high flow rate. Peptides were eluted through a reverse-phase capillary column (75 μm inner diameter x 150 mm) containing Symmetry C18 100 Å medium (Waters) that was self-packed using a high-pressure packing device (Proxeon Biosystems).

#### Database searching

All MS/MS samples were analyzed using Mascot (Matrix Science; version 2.2.04) and X! Tandem (The GPM, thegpm.org; version CYCLONE (2010.12.01.1). Mascot and X! Tandem were searched with a fragment ion mass tolerance of 0.020 Da and a parent ion tolerance of 10.0 PPM. The UniprotHuman_2013_08 database (88,378 entries) and a custom database containing the amino acid sequence of human GPR84-eYFP were searched. Carbamidomethyl of cysteine was specified as a fixed modification, Glu->pyro-Glu of the N terminus, ammonia loss of the N terminus, gln->pyro-Glu of the N terminus, oxidation of methionine and phosphorylation of serine, threonine, and tyrosine were specified in X! Tandem as variable modifications. Oxidation of methionine and phosphorylation of serine, threonine, and tyrosine were specified in Mascot as variable modifications.

#### Criteria for peptide identification

Scaffold (version Scaffold_4.8.7, Proteome Software Inc) was used to validate MS/MS-based peptide and protein identifications. Peptide identifications were accepted if they could be established at greater than 20.0% probability. Peptide Probabilities from X! Tandem and Mascot were assigned by the Scaffold Local FDR algorithm. Peptide Probabilities from X! Tandem were assigned by the Peptide Prophet algorithm (41) with Scaffold delta-mass correction. Protein identifications were accepted if they could be established at greater than 95.0% probability and contained at least two identified peptides. The mass spectrometry proteomics data have been deposited to the ProteomeXchange Consortium *via* the PRIDE partner repository (42) with the dataset identifiers PXD031252 and 10.6019/PXD031252.

### Molecular modeling

2-HTP, 6-OAU, and DL-175 were docked into the previously published hybrid template homology model (24) and the recently released AlphaFold model of the human GPR84 (37) using a standard precision docking protocol available in the Glide module of Schrodinger software (2020-1) (43, 44). The protein structures were prepared with the Protein Preparation Wizard, whereas the agonists were generated with the Ligand Preparation module of Schrodinger software. The docking box was centered based on Tyr^69^, Phe^96^, Leu^99^, Phe^101^, Arg^172^, Phe^335^, and Trp^360^ residues. The poses were selected based on the Glide docking score and taking into consideration experimental information. Minimization of docking complexes was performed using the MacroModel module of Schrodinger software. A default protocol with 1000 steps of minimization in implicit solvent was used to obtain the final complex. The OPLS_2005 force field was used in MacroModel calculations. The images for Figures 11 and 12 were created in Maestro 2020-1.

### Data analysis

All data are presented as means ± SEM of at least three independent experiments. Data analysis and curve fitting was carried out using the GraphPad Prism software package version 8 (GraphPad). For functional assays the concentration–response data were plotted on a log axis, with the untreated vehicle control plotted at 1 log unit lower than the lowest ligand concentration and fitted to a three-parameter sigmoidal curve with the Hill slope constrained to equal 1. To perform the statistical analysis of curve parameters, data from multiple experiments were fitted independently and the resulting curve fit values were analyzed with indicated tests. Antagonism experiments carried out with multiple defined concentrations of antagonist were fit to a global Gaddum/Schild EC_50_ shift equation to estimate pA_2_ values for the antagonist.

## Data availability

The mass spectrometry proteomics data have been deposited to the ProteomeXchange Consortium *via* the PRIDE partner repository with the dataset identifier PXD031252 and 10.6019/PXD031252. All other data are freely available upon request to the correspondent author graeme.milligan@glasgow.ac.uk.

## Conflict of interest

S. S. is a founder of 7TMAntibodies; F. N. is an employee of 7TMAntibodies; the other authors declare no conflict of interests.
